# Continuous Feeding Reduces the Generation of Metabolic Byproducts and Increases Antibodies Expression in Chinese Hamster Ovary-K1 Cells

**DOI:** 10.3390/life11090945

**Published:** 2021-09-10

**Authors:** Shang Xiao, Waqas Ahmed, Ali Mohsin, Meijin Guo

**Affiliations:** State Key Laboratory of Bioreactor Engineering, East China University of Science and Technology, Shanghai 200237, China; shang.xiao@lyvgen.com (S.X.); waqasahmed@mail.ecust.edu.cn (W.A.); alimohsin@ecust.edu.cn (A.M.)

**Keywords:** Chinese hamster ovary (CHO), continuous feeding, lactate accumulation, NH_4_^+^ metabolism, high mannose content, osmolality

## Abstract

Chinese hamster ovary (CHO) cells are the most important host system used for monoclonal antibody (mAb) expression. Moreover, the fed-batch culture mode is the most widely used method to increase mAb expression in CHO cells by increasing the amount of feed. However, a high amount of culture feed results in the production of metabolic byproducts. In this work, we used a continuous feeding strategy to reduce metabolic byproducts and improve mouse–human chimeric anti-epidermal growth factor receptor vIII (EGFRvIII) antibody C12 expression in Chinese hamster ovary-K1 cells. Moreover, the effects of the feeding strategy on the cell culture and monoclonal antibody production were evaluated in chemically defined suspension cultures of recombinant CHO-K1 cells. Compared with bolus feeding methods, the continuous feeding method did not have any advantages when the feeding amount was low, but with a high feeding amount, the continuous feeding method significantly reduced the concentrations of lactate and NH_4_^+^ in the later culture stage. At the end of the culture stage, compared with bolus feeding methods, the lactate and NH_4_^+^ concentrations under the continuous feeding mode were reduced by approximately 45% and 80%, respectively. In addition, the antibody C12 expression level was also increased by almost 10%. Compared to the bolus feeding method, the antibody C12 produced by the continuous feeding method had a lower content of high-mannose glycoforms. Further analysis found that the osmolality of the continuous feeding method was lower than that of the typical fed-batch bolus feeding method. Conclusively, these results indicate that the continuous feeding method is very useful for reducing metabolic byproducts and achieving higher levels of mAb production.

## 1. Introduction

Since the approval of the first therapeutic antibody drug orthoclone OKT3 by the USA Food and Drug Administration (FDA) in 1986, antibody drugs have become the fastest growing sector of drug development in the world after more than 30 years of technological breakthroughs [1,2]. Currently, Chinese hamster ovary (CHO) cells are the major host cell to produce high quality and quantity biopharmaceuticals due to their ability to produce antibodies with similar posttranslational modifications as those of human IgGs, and they are easy to adapt to serum-free suspension culture [3,4]. Nearly 70% of the postlaunch therapeutic proteins are expressed by CHO cells [4,5,6]. In recent years, these biological agents have often been used by patients in large quantities, and their global demand is rapidly increasing. Therefore, it is very urgent to increase the protein expression level of CHO cells and reduce the cost of production.

Fed-batch culture is the most common and mature CHO cell culturing process due to its simple operation and high production [7], but this process tends to accumulate large amounts of metabolic byproducts, such as lactate and ammonium salt (NH_4_^+^), in the later stages of culture [8,9]. Previously, many studies indicated that high concentrations of lactate and NH_4_^+^ not only are unfavorable for cell growth and antibody expression but can also cause instability in the cell culture process [10,11,12,13]. Therefore, it is very important to control metabolic byproducts at low concentration levels; however, controlling the lactate and NH_4_^+^ concentrations is a major challenge in cell culture technology [13]. For the fed-batch process, the protein expression level is usually increased by increasing the amount of concentrated nutrient feed. However, a high feeding amount will cause more cell metabolism byproducts, which will eventually affect the stability of the process and even reduce the protein expression. Therefore, solving a series of problems caused by high feeding amounts is very important to reduce the cost of biopharmaceuticals.

For the fed-batch process, there are numerous studies and reviews describing the effects of process parameters such as the temperature, pH, and other factors on antibody expression and quality attributes [14,15,16]. However, so far, no study has reported that the feeding method had a huge impact on CHO cell culture. Usually, a fed-batch culture is fed once per day or multiple days [17]. However, under specific conditions of a high feeding amount, this bolus feeding method will surely generate high levels of metabolic byproducts. In this study, we evaluated the effects of different feeding amounts and feeding methods on process performance and antibody quality to determine the effects of the new feeding method (continuous feeding), which ultimately reduces the metabolic byproducts generated by high feeding. The experiments were conducted in a 7 L bioreactor (Applikon, the Netherlands). Moreover, we detected the relevant in-process indicators, antibody concentration and glycosylation at the end of the culture stage. The information obtained in this study will be of great significance for improving protein expression and quality with high feeding amounts.

## 2. Materials and Methods

### 2.1. Cell Lines and Media

The two glutamine synthetase (GS) CHO cell lines used in this study to produce EGFRvIII antibody C12 were generated from CHO-K1 cells, as described previously [18], and were adapted to suspension culture, with have the ability to grow in serum-free medium. Briefly, by co-transfecting the desired EGFRvIII antibody C12 gene with GS gene into CHO-K1 cells, adding MSX screening, and performing subcloning, stable CHO cell lines A and B were obtained, which produced human mAb C12 A and mAb C12 B, respectively. Both C12 A and C12 B have a conserved N-glycosylation site at the N297 position.

The basal media and feeding media used in this study were chemically defined serum-free media. The basal medium was Dynamis, which was purchased from Gibco. The feeding media were Cellboost 7a (Cb7a) and Cellboost 7b (Cb7b), which were purchased from GE Healthcare. Each Cb7b supplement was 1/10 of Cb7a. Glucose concentration was tested off-line once a day from day 3, and then 400 g/L glucose stock solution was feed to adjust the glucose concentration to 8 g/L during the cell culture process to supply sufficient amounts of an energy source and cellular components.

### 2.2. Process Conditions

Experiments were performed in a 7 L bioreactor (Applikon, the Netherlands) with a maximum working volume of 5.6 L. In our study, the initial culture volume was 3 L. The initial cell concentration was 0.5 × 10^6^ cells/mL, the pH was controlled at 7.2 ± 0.1 with 7.5% sodium bicarbonate and CO_2_, and the dissolved oxygen (DO) was controlled at 40% saturation by oxygen flow. The temperature was maintained at 37 °C, and feeding was started on day 3.

In this study, we conducted nine experiments for each cell line, as summarized in Table 1.

The culture durations of cell lines A and B were 17 days and 15 days, respectively. All the processes were performed on day 3. Processes 1, 2, and 3 were fed once every two days; processes 4, 5, and 6 were daily feeding processes; and processes 7, 8, and 9 were continuous feeding processes. The feeding amounts of processes 1, 4, and 7 were the same; those of processes 2, 5, and 8 were the same; and those of processes 3, 6, and 9 were the same.

### 2.3. Cells, Metabolites and Osmolality Analysis

Cell culture samples were taken from each bioreactor every day during the entire culture duration. Viable cell density (VCD) and viability were measured in an automated cell counting device (Vi-cell, Beckman, CA, USA) by trypan blue staining. Lactate, NH_4_^+^, glucose, and glutamate were monitored using a Nova Biomedical 400 Analyzer (Nova Biomedical, Waltham, MA, USA). When these parameters were below the detection limit, this study defaulted to 0. Osmolality was tested by a Model 3250 Osmometer (Advanced, Norwood, MA, USA).

Supernatant samples were stored at −20 °C. At the end of the experiments, frozen cell-free supernatant samples were thawed and collectively submitted for yield and free amino acid analysis by high-performance liquid chromatography (HPLC) (HP1100, Agilent, CA, USA).

### 2.4. Antibody Analysis by HPLC

After centrifuged, the cell culture supernatant samples were injected into the HPLC system (Agilent, CA, USA) equipped with UV detection at 280 nm. The column was TSKgel Protein A-5PW 4.6 mm × 35 mm, 20 μm (Tosoh Yamaguchi, Japan). The flow rate was 1 mL/min. The gradient method using mobile phase 50 mM sodium phosphate/150 mM sodium chloride and 100 mM glycine/150 mM sodium chloride was used to elute each sample every 8.0 min.

### 2.5. Physicochemical Analysis

Cell supernatants were collected and purified on days 15 and 17 by a protein A column.

For size variant analysis, the samples were analyzed by a TSK G3000SWXL column 7.8 mm × 300 mm, 5 μm (Tosoh, Yamaguchi, Japan) with a mobile phase buffer (50 mM NaH_2_PO_4_, 250 mM NaCl, pH 6.8) at a constant flow rate of 0.5 mL/min.

For size variant analysis, CE-SDS was performed under nonreducing conditions for analysis of purity/impurities. A Beckman Coulter, PA 800 capillary electrophoresis system was used, with an effective length of 30.2 cm and a 50 mm I.D. bare-fused silica capillary.

For charge variant analysis, the samples were analyzed by Propac WCX10 4 mm × 250 mm, 5 μm (Thermo, Waltham, MA, USA). Gradient elution was performed at a constant flow rate of 0.8 mL/min.

For oligosaccharide profile analysis, N-linked glycans were first enzymatically released from the antibody with peptide-N-glycosidase F (pNGase F), labeled with 2-aminobenzamide, and subsequently analyzed by ultra-performance liquid chromatography (UPLC) with fluorescence detection.

### 2.6. Statistical Analysis

SPSS 19 software was used to perform statistical analysis. All statistical values are presented as means ± standard deviation (SD). The data shown in the figures are representative of experiments performed in triplicate. Evaluation of statistical significance (*p* < 0.05) was calculated according to the *t*-test.

## 3. Results and Discussion

To study the effects of feeding strategies on CHO cell growth and C12 production, fed-batch cultures were initiated at a cell concentration of 0.5 × 10^6^ cells/mL in 7 L bioreactors, as shown in Table 1. These experiments were performed on two cell lines, and to better analyze the impact of the feeding methods, three feeding methods were evaluated: feeding once every two days, feeding once a day, and continuous feeding. Moreover, each of the three feeding methods further consisted of three different feeding amounts, as shown in Table 1.

### 3.1. Effects of Three Different Feeding Processes on CHO Cell Growth

**Figure 1:**
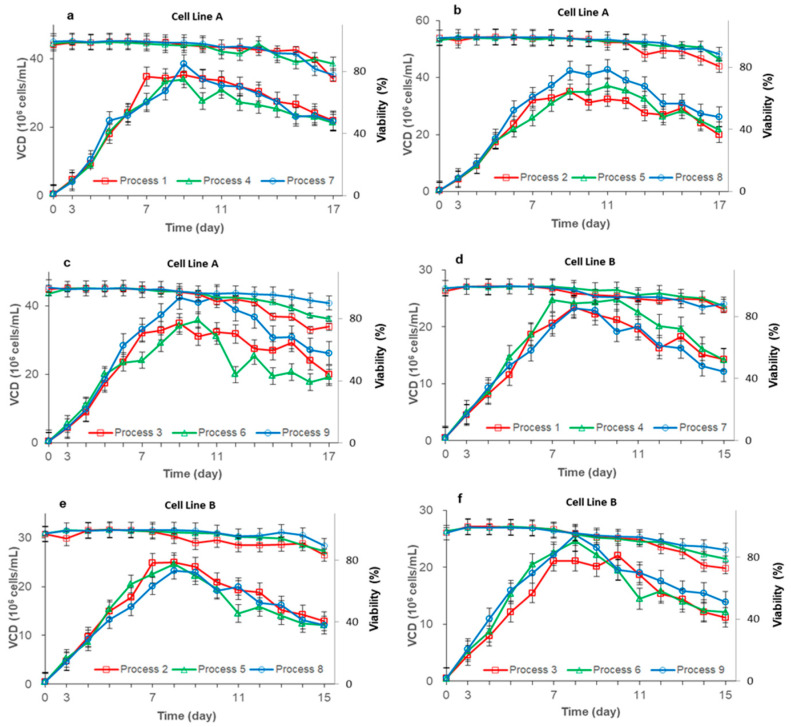
Effect of the feeding strategy on the VCD and viability. (**a**–**c**) compare the effects of different feeding methods on cell line A when the feeding amount is low (**a**), medium (**b**), and high (**c**), respectively. d, e, and f compare the effects of different feeding methods on cell line B when the feeding amount is low (**d**), medium (**e**) and high (**f**), respectively. Data indicate means ± standard errors of three replicates.

The data of the three different feeding processes with the same feeding amount were compared (Figure 1). At a low feeding amount (Figure 1a,d), there were no significant difference in cell growth for both cell lines A and B among the three different feeding methods; these may be because the CHO cells did not produce many metabolic byproducts when the feeding amount was low [19], so the cell growth was similar under the three feeding methods. At a medium and high feeding amount(Figure 1b,c,e,f), for cell line A, continuous feeding showed better growth, a higher peak VCD and increased viability maintenance. According to our limited knowledge, this is the first report that the continuous feeding process can increase the maximum viable cell density. These results may be related to the effect of nutrient levels and osmolality on the cell growth cycle [20]. However, this result did not reproduce on cell line B. 

When feeding was performed every two days or daily, a high feeding amount resulted in a lower VCD and viability in the late cultivation time period. This result may be due to greater dilution and metabolic by-products caused by higher feed amount [12,21]. Within the scope of our research, we did not find that the continuous feeding process with a high feeding amount has a lower late-cultivation viability, which indicates that the continuous feeding process increases the threshold of feeding amount that the process can tolerate.

### 3.2. Effects of Feeding Strategies on Cell Metabolism

**Figure 2:**
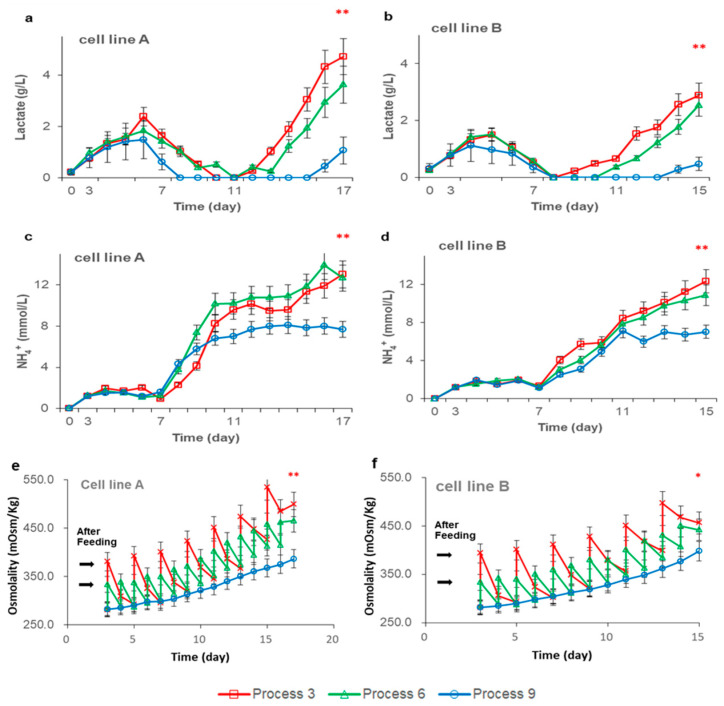
Effects of the feeding strategy on cell metabolism. (**a**,**b**) are comparisons between the effects of different feeding methods on the concentration of lactate with a high feeding amount in cell lines A and B, respectively. (**c**,**d**) are comparisons between the effects of different feeding methods on the concentration of NH_4_^+^ with a high feeding amount in cell lines A and B, respectively. (**e**,**f**) are comparisons between the effects of different processes on the osmolality in cell lines A and B, respectively. Data indicate means ± standard errors of three replicates. Statistically significant differences are defined as *p* < 0.05. Statistical significance * for *p* < 0.05, ** for *p* < 0.01.

It is often necessary to increase the amount of feeding to obtain a higher yield when using the fed-batch culture process, but high feeding may lead to an increase in lactate, NH_4_^+^, and osmolality, which are the key byproducts of CHO cell metabolim [21]. For lactate-depleting CHO cells, lactate is usually produced first and then consumed [22]. For CHO cells with the GS system, NH_4_^+^ levels is accumulated slowly in the later stages. As shown in Figure 2, under high feeding conditions, continuous feeding produced lower levels of lactate, NH_4_^+^, and osmolality at the late stage of the culture. The reason is possibly that traditional bolus feeding methods can introduce high concentrations of nutrients within a short period of time, which will ultimately induce more metabolic byproducts [21]. In addition, after feeding, the osmolality will rise rapidly and greatly, and such a high osmolality will also induce cells to produce more metabolic byproduct. The continuous feeding process can control the concentration of nutrients in the bioreactor in an appropriate range, so it can reduce the production of metabolic byproducts such as lactate. Previously, it was reported that a high concentration of lactate, NH_4_^+^, or osmolality would affect the maintenance of cell viability and the expression of antibodies [23,24,25,26]. The continuous feeding process has lower metabolic byproduct generation, which may be the reason for the higher VCD and viability at the late stage of the culture when using the continuous feeding process.

**Figure 3:**
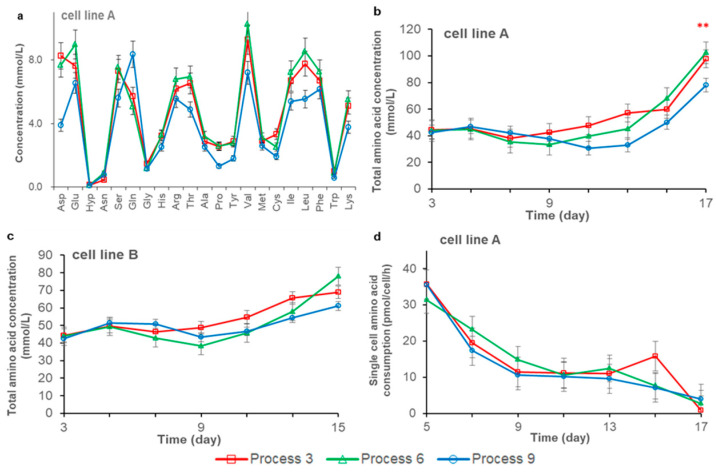
Amino acid metabolism. (**a**) shows the comparison of the concentration of each residual free amino acid in cell line A on day 17; (**b**) shows the comparison of the total concentration of residual free amino acids in cell line A from day 3 to day 17; (**c**) shows the comparison of the total concentration of residual free amino acids in cell line B from day 3 to day 15; (**d**) shows the single cell amino acid consumption in cell line A from day 5 to day 17; Data indicate means ± standard errors of three replicates. Statistically significant differences are defined as *p* < 0.05. Statistical significance ** for *p* < 0.01.

To verify our speculation that continuous feeding can lower the level of nutrients in the culture system, the residual amino acid levels of different processes under three high feeding amounts were tested and analyzed with cell line A and B. The residual amino acid concentrations were added to obtain the total amino acid concentration, as shown in Figure 3b,c. For cell line A, the total residual amino acid concentration in the continuous feeding process was the lowest, which was only 80% of daily feeding and feeding once every two days. This may have contributed to cell line A producing lower lactate and NH_4_^+^ levels when using a continuous feeding process. The lower amino acid concentration would further reduce the osmolality of the culture environment (Figure 2e,f). Low osmolality and substrate concentration would further reduce the production of metabolic byproducts (lactate and NH_4_^+^) [27]. The continuous feeding process introduced lower concentrations of aspartic acid (Asp), glutamate (Glu), serine (Ser), threonine (Thr), proline (Pro), valine (Val), isoleucine (Ile), leucine (Leu), lysine (Lys), and the sum of cysteine and cystine (Cys) at the end of the cultivation, but the glutamine (Gln) concentration was higher, and there are no obvious difference in other amino acids (Figure 3a). The low substrate concentration reduced the rate of lactate and NH_4_^+^ generation, so as to maintain a higher cell viability [21]. At the same time, higher cell viability can synthesize the Gln enzyme more efficiently; therefore, more NH_4_^+^ and Glu can be synthesized into Gln. This is also consistent with the higher Gln levels in the supernatant of the continuous feeding process.

The single-cell amino acid consumption rate of cell line A was further analyzed (Figure 3d), and there was no significant difference between different feeding processes. However, the continuous feeding process obtained a higher VCD, which consumes more amino acids, so the residual amino acid concentration will be lower. The cell line B has no obvious difference in VCD between different feeding processes, so there is no significant difference in total residual amino acid concentration between different feeding processes.

These results indicate that the continuous feeding process cannot increase the amino acid consumption rate of single cells. As to whether the residual amino acid concentration can be reduced is related to the cell density, and the continuous feeding process may not necessarily reduce the residual amino acid concentration. Combined with Figure 2e,f, the results show that compared with continuous feeding, traditional bolus feeding has higher amounts of metabolic by-products, which may be because bolus feeding can increase osmolality and nutrient concentration to a high level in a short time after feeding, which induces higher amounts of metabolic by-products.

### 3.3. Effects of Feeding Strategies on Antibody Expression and Quality

**Figure 4:**
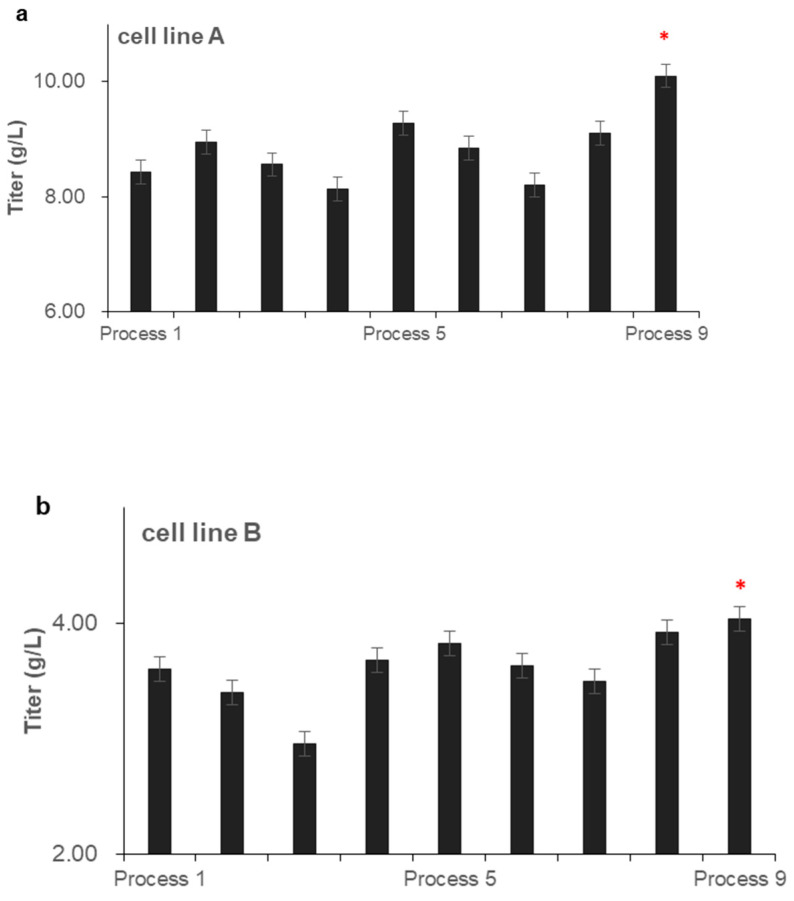
Effect of the feeding amount and strategy on antibody C12 expression. (**a**) shows the effects of different processes on C12 expressed by cell line A. (**b**) shows the effects of different processes on C12 expressed by cell line B. Data indicate means ± standard errors of three replicates. Statistically significant differences are defined as *p* < 0.05. Statistical significance * for *p* < 0.05.

The yields of all processes at the end of culture are shown in Figure 4. For cell line A, the yield of feeding once every two days process and the daily feeding process showed an increasing trend first, followed by a decline as the feeding amount increased. However, the yield of the continuous feeding process was always rising as the feeding amount increased. With the low feeding amount, there were no differences in the yield between the three kinds of feeding processes. With the medium feeding amount, the yield of feeding once every two days process was the lowest, and there was no difference between the daily feeding and continuous feeding processes. With the high feeding amount, the continuous feeding process obtained the highest yield, exceeding 10 g/L, and the feeding once every two days process obtained the lowest yield, at 8.56 g/L. For cell line B, the experimental results were similar to cell line A, the continuous feeding process obtained the highest yield, exceeding 4.0 g/L. 

Although the easiest way to increase antibody expression is to increase the amount of feed, this strategy will quickly reach the threshold for the traditional bolus feeding process because of lacate, NH4^+^, or osmolality [28]. However, the continuous feeding process can further increase the threshold of the feeding amount, which will further increase antibody expression.

**Figure 5:**
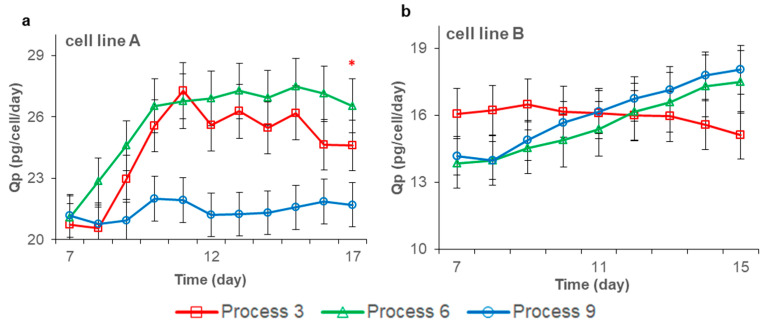
The effect of the feeding amount strategy on Qp. (**a**) compares the effects of different processes on the Qp of cell line A. (**b**) compares the effects of different processes on the Qp of cell line B. Data indicate means ± standard errors of three replicates. Statistically significant differences are defined as *p* < 0.05. Statistical significance * for *p* < 0.05.

Further analysis of the specific production rates (Qp) obtained by the different processes (Figure 5. For cell line B, there was no significant difference in the Qp between different processes (*p* > 0.05). However, for cell line A, the Qp of continuous feeding was lower than that of the other processes (*p* < 0.05), mainly due to the lower nutrient concentration obtained by continuous feeding [15]. It may also be related to the cell growth cycle [29], and we assume that the continuous feeding process may obtain a lower G0/G1 ratio. Therefore, we believe that the Qp of continuous feeding process has the possibility of further improvement.

The antibodies of the three processes with high feeding amounts for cell lines A and B were purified by protein A in one step, and the antibodies were used to quality analysis.

IgG antibodies produced in CHO cells generally contain low levels of high-mannose glycoforms (Man5–9), typically below 5%, with the most common glycans being the complex glycoforms G0F, G1F, and G2F. High levels of high mannose glycoforms on therapeutic antibodies could be a concern due to uncertainty about their impact on clearance, immunogenicity and efficacy [30,31,32]. Therefore, we need to control the high-mannose glycoform content of antibodies. Antibodies charge variants may have a significant impact on the pharmacokinetics and pharmacodynamics of the antibody [33,34,35]; therefore, it needs to be strictly controlled.

By testing the quality of the antibodies obtained from the three processes, we found that the high-mannose glycoform level of the continuous feeding processes was decreased (*p* < 0.05) (Table 2 and Table 3). Pacis et al. reported that a high osmolality level could increase the high-mannose glycoform content [30]. Since continuous feeding processes have a lower osmolality level (as shown in Figure 2), this may explain why the continuous feeding process had a decreased high-mannose glycoform content. There was no significant difference in the other quality attributes.

Currently, the biopharmaceutical industry is developing rapidly; however, due to the low expression of mammalian cells and the high drug dosages used by patients, the production capacity and production costs of biopharmaceuticals have come to reprsent a significant bottleneck for the popularity of biopharmaceuticals. Therefore, it is very important to improve the expression ability of mammalian cells and reduce the production cost of biological drugs. Currently, the most widely used mammalian cell type for biopharmaceutical production is CHO cells, and the most popular CHO cell culture process is the fed-batch process because the fed-batch process has the advantages of low production cost, simple operation, and stable processes [36]. However, the fed-batch process also has the disadvantages of low production efficiency and easy accumulation of metabolic byproducts in the later stages of the culture [21]. The accumulation of metabolic byproducts in the later culture stages may reduce the efficiency of protein production and cause process instability [37]. To improve the production efficiency of the fed-batch culture process, the simplest method is to increase the feeding amount, but increasing the feeding amount will bring more metabolic byproducts, which may prevent the increase of antibody expression. Therefore, solving the high metabolic byproduct caused by the high feeding amount may further improve the protein expression ability of CHO cells.

In our experiments, the results showed that changing the feeding methods may reduce the concentration of high metabolic byproducts brought by high feeding amounts. Therefore, to investigate the effect of the feeding strategy on the CHO cell culture and antibody quality more comprehensively, a comparative studies were conducted on two cell lines and three different feeding levels. The experimental results showed that at low feeding levels, there was no significant difference between the three different feeding processes, and the gap between the three feeding processes slowly emerged as the amount of feeding increased. For cell line A, the continuous feeding process can obtain a higher peak VCD and yield with a high feeding amount. However, for cell line B, there was no significant difference in the peak VCD obtained under different feeding processes, but continuous feeding could maintain a higher peak VCD and viability at the end of culture, such that the final antibody expression of the continuous feeding process was 5~15% higher than that of the next-day feeding and daily feeding processes. These results indicate that the continuous feeding process can better maintain cell viability at the later stage of culture than the other feeding processes.

Both cell lines A and B results showed that continuous feeding can reduce the levels of lactate and NH_4_^+^ significantly in the late cultivation period. Detecting and analyzing of amino acids in the supernatant of the three culture processes of cell line A and B, the results showed that the concentration of residual free amino acids before feeding is not the cause of more metabolic by-products. Compared with continuous feeding, traditional bolus feeding has higher metabolic by-products, which may be due to bolus feeding can increased osmolality and nutrient concentration to a high level in a short time after feeding which inducing more metabolic by-products. A lower amino acid substrate concentration would reduce the energy supply of amino acids through oxidation, thereby reducing the rate of NH_4_^+^ and lactate production.. The continuous feeding process obtained a lower osmolality, which caused a lower high-mannose glycoform content in the antibodies, so the continuous feeding process improved the quality of the antibodies. Thus, there were no significant differences in other quality attributes between the three kinds of fed-batch processes.

## 4. Conclusions

In conclusion, we found that when the amount of feeding was low, the continuous feeding process did not have a significant effect because the nutrient level was in a lower range. However, when the feeding amount was increased (high), compared with traditional bolus feeding (feeding once a day or multiple days), continuous feeding could reduce the cell metabolic byproducts and osmolality and improve the CHO cell viability and the VCD in the later period of cultivation. Finally, the continuous feeding process increased the threshold of the feeding amount that the CHO cells could tolerate, thereby increasing the antibody expression by approximately 10%/ At the same time, the level of metabolic byproducts in the later stage of the culture were reduced significantly; thus, cell culture process can become more stable. Compared with the traditional bolus feeding process, the continuous feeding process produced a lower metabolic byproduct concentration and osmolality, which resulted in a lower high-mannose glycoform content, thereby improving the antibody quality of the antibodies obtained under the continuous feeding process. To our knowledge, this is the first report indicating that compared to bolus feeding processes, the continuous feeding process reduces the concentration of metabolic byproducts, the high-mannose glycoform content and increases the expression of CHO cells at high feeding rates. In short, these results will surely provide a good foundation for the biopharmaceutical industry to increase their protein production capacity and cell culture process stability, which will ultimately reduce their production costs.

## Figures and Tables

**Table 1:** Summary of the processes used in this study.

Process	Feed Methods	Total CB7a Feeding Amount (V/V%) ①		Feeding Strategy	
		Cell Line A	Cell Line B	Cell Line A	Cell Line B
Process 1	Once every two days	28	24	120 mL each time from Day 3	
Process 2	Once every two days	35	30	150 mL each time from Day 3	
Process 3	Once every two days	42	36	180 mL each time from Day 3	
Process 4	Daily feeding	28	24	60 mL each time from Day 3	
Process 5	Daily feeding	35	30	75 mL each time from Day 3	
Process 6	Daily feeding	42	36	90 mL each time from Day 3	
Process 7	Continuous feeding ②	28	24	Starting from the third day, the on-time of the pump was set to 2S; the off-time was set to 738S	
Process 8	Continuous feeding	35	30	Starting from the third day, the on-time of the pump was set to 2S; the off-time was set to 590S	
Process 9	Continuous feeding	42	36	Starting from the third day, the on-time of the pump was set to 2S; the off-time was set to 491S	

① *V*/*V* means total feeding volume/initial culture volume. ② The pump speed was 15.4 mL/min and continuous feeding can be achieved by setting the on- and off-times of the bioreactor pump.

**Table 2:** The quality of C12 expressed by cell line A grown with high feeding amounts.

	Quality	Process 3	Process 6	Process 9
Cell line A	SEC purity (%)	98.79 ± 0.43	99.05 ± 0.48	98.88 ± 0.54
	CEX AV (%)	24.60 ± 3.21	24.75 ± 2.88	22.70 ± 3.01
	CEX MP (%)	66.08 ± 5.25	67.16 ± 4.72	68.23 ± 3.76
	CEX BV (%)	9.32 ± 2.23	8.09 ± 1.85	9.06 ± 1.54
	G2F (%)	0.27 ± 0.15	0.13 ± 0.13	0.33 ± 0.18
	G1F (%)	7.80 ± 1.26	8.30 ± 1.83	7.53 ± 1.37
	G0F (%)	86.63 ± 9.19	88.17 ± 8.38	89.37 ± 9.02
	High-mannose (%)	2.15 ± 0.47	1.39 ± 0.43	0.88 ± 0.24
	NR-CE (%)	96.12 ± 1.13	96.85 ± 1.56	96.52 ± 1.45

**Table 3:** The quality of C12 expressed by cell line B grown with high feeding amounts.

	Quality	Process 3	Process 6	Process 9
Cell line B	SEC purity (%)	97.75 ± 0.54	97.45 ± 0.50	98.05 ± 0.59
	CEX AV (%)	18.24 ± 2.34	19.23 ± 2.07	18.01 ± 1.34
	CEX MP (%)	72.55 ± 4.54	72.01 ± 3.54	72.26 ± 3.29
	CEX BV (%)	9.21 ± 1.47	8.76 ± 1.09	9.73 ± 1.21
	G2F (%)	2.17 ± 1.38	1.89 ± 1.53	2.23 ± 1.77
	G1F (%)	11.20 ± 1.88	13.15 ± 2.01	12.45 ± 2.34
	G0F (%)	78.28 ± 8.78	77.58 ± 7.56	80.30 ± 9.57
	High-mannose (%)	4.35 ± 1.43	3.38 ± 0.76	1.02 ± 0.35
	NR-CE (%)	95.64 ± 1.02	95.43 ± 0.87	95.78 ± 0.93

AV: acid variety. MP: main peak. BV: alkaline variety. G2: contains two galactose molecules. G1: contains one galactose molecule. G0: contains no galactose molecule. F: fucose. NR-CE: nonreduced CE. Values of each parameter are reported as average ± standard deviation (*n* = 3).

## Data Availability

The data presented in this study are available in this article.
